# Abnormal Head Position in Infantile Nystagmus Syndrome

**DOI:** 10.5402/2011/594848

**Published:** 2012-01-03

**Authors:** Susana Noval, Mar González-Manrique, José María Rodríguez-Del Valle, José María Rodríguez-Sánchez

**Affiliations:** ^1^Hospital de La Paz, Universidad Autónoma de Madrid, IdiPaz, 28029 Madrid, Spain; ^2^Hospital de Móstoles, Universidad Rey Juan Carlos, 28935 Madrid, Spain; ^3^Hospital 12 de Octubre, Universidad Complutense, 28041 Madrid, Spain; ^4^Hospital Ramón y Cajal, Universidad de Alcalá de Henares, 28034 Madrid, Spain

## Abstract

Infantile nystagmus is an involuntary, bilateral, conjugate, and rhythmic oscillation of the eyes which is present at birth or develops within the first 6 months of life. It may be pendular or jerk-like and, its intensity usually increases in lateral gaze, decreasing with convergence. Up to 64% of all patients with nystagmus also present strabismus, and even more patients have an abnormal head position. The abnormal head positions are more often horizontal, but they may also be vertical or take the form of a tilt, even though the nystagmus itself is horizontal. The aim of this article is to review available information about the origin and treatment of the abnormal head position associated to nystagmus, and to describe our treatment strategies.

## 1. Introduction

Infantile nystagmus is an involuntary, bilateral, conjugate, and rhythmic oscillation of the eyes which is present at birth or develops within the first 6 months of life [1]. It may be associated with an afferent visual defect such as albinism, congenital cataract, retinal dystrophy, or optic atrophy; or it may appear without visual or neurological impairment, in which case it is termed idiopathic [2, 3]. In Abadi and Bjerre's series with 224 subjects with infantile nystagmus, most of them were classified as idiopathic (62%), 28% as albinos and only 10% exhibited other ocular pathologies [1]. Family history was documented in one third of the patients; the most common pattern of inheritance was dominant autosomal (70%), followed by X-linked (26%), and autosomal recessive [1].

Congenital nystagmus is one of the main causes of torticollis [4]. Our purpose is to describe the abnormal head position that can be present in many patients with nystagmus from a clinical and a surgical point of view. However, some concepts have to be previously explained.

## 2. Concepts and Definitions

**(i) Null zone**

The position of the gaze in which the oscillation is minimum or absent [1]. The multiple factors that influence the null zone might explain why the anomalous head posture has been observed to change with time.

**(ii) Foveation periods**

The length of time during a nystagmus cycle when the eyes are more stable. This period of time corresponds to maximum afferent attention, when the patient sees more clearly. Foveation time is a better indicator of visual function than the intensity of the oscillation [5].

**(iii) Intensity**

The intensity of nystagmus can be measured by multiplying the amplitude (size) by the frequency (cycles per second) of the movements.

**(iv) Periodic alternating nystagmus**

Nystagmus in which the direction of the slow phase changes periodically, producing temporal shifts in the gaze angle at which the null zone occurs [1]. The periodicity of direction reversal is less regular in congenital than in acquired cases.

**(v) Latent nystagmus**

is defined as a nystagmus triggered by occlusion

**(vi) Pendular and jerk waveforms**

Nystagmus is classified into pendular and jerk waveforms. The former is characterized by an oscillation that has similar velocities in each direction (sinusoidal movements), whereas the latter is characterized by markedly unequal velocities with a slow error-producing component followed by a fast error-correcting saccade [6].

## 3. Clinical Features [1]

- Horizontal oscillations, even in up gaze, with a frequent torsional component, which is usually evident in recordings, but not always clinically obvious.
- Pendular or jerk nystagmus. Growth and development of the visual sensory system evoke evolution of waveforms during early infancy from pendular to jerk-type nystagmus [5].
- Intensity increases on lateral gaze and the direction is right-beating in right gaze and left-beating in left gaze.
- Infantile oscillations become worse with fixation attempts, fatigue, stress, and attention [1].
- It may decrease or disappear with convergence, which results in better near visual acuity compared to distance visual acuity.
- With-the-rule astigmatism.

Up to 64% of all patients have strabismus, mainly horizontal tropias, with a roughly equal distribution between eso and exo deviations. Differences among idiopathic and secondary cases have been described for ocular alignment and visual acuity. Strabismus is more frequent among albinos (90%) and visual acuity and stereoacuity are significantly higher in idiopathic cases than in patients with albinism or ocular anomalies [1].

### 3.1. Abnormal Head Position

In most patients with infantile nystagmus, the head position corresponds roughly to the null zone. Abadi et al. studied the head position in 143 subjects with infantile nystagmus and found that 73% had spatial null zones within plus or minus 10° of the primary position [1]. However, Spielmann studied 47 patients with manifest congenital nystagmus and found no abnormal head posture in only 3 patients (6%) [7]. Hertle et al. studied 27 children aged from 3 to 6.5 months and only 5 (19%) had an anomalous head posture at that time. Abnormal head positions when present were related to a null zone other than primary position in most cases, or to an absence of a clear null position [5]. This latter study suggested that there may be changes in the head posture during the first years of life.

Anomalous head position is more often horizontal, but it may also be vertical or take the form of a tilt even though the nystagmus itself is horizontal [8]. In Spielmann's study, horizontal torticollis was present in 12 patients (26%), alternating head turn in 6 (13%), a blocking convergence in 5 (10%), a vertical abnormal posture in 7 (15%), a pure head tilt in one case, and a mixed head position in 13 patients (2%) [7].

### 3.2. Convergence

Infantile nystagmus often decreases during convergence, which results in a near visual acuity that is better than the distance acuity. Parents may report that children view objects at a very close distance. Abadi et al. examined the effect of convergence on the intensity of the nystagmus in 117 subjects, of which 44% showed a decrease in the nystagmus intensity during near fixation compared with distance fixation [1]. They were evenly distributed among idiopathic, albino, and ocular anomaly groups. Accomodative behavior in congenital nystagmus has been related to an increased depth-of-focus [9]. Spielmann considered that fusional convergence may decrease the nystagmus in those cases associated with exophoria [7].

## 4. Examination

Family and personal *histories* should be investigated and followed by a comprehensive ocular examination to rule out ocular pathologies that might be the cuase of the nystagmus. Secondary cases may need a specific treatment, like congenital cataracts, or further explorations, like optic nerve atrophies (in which an MRI should be performed).

**Figure 1:**
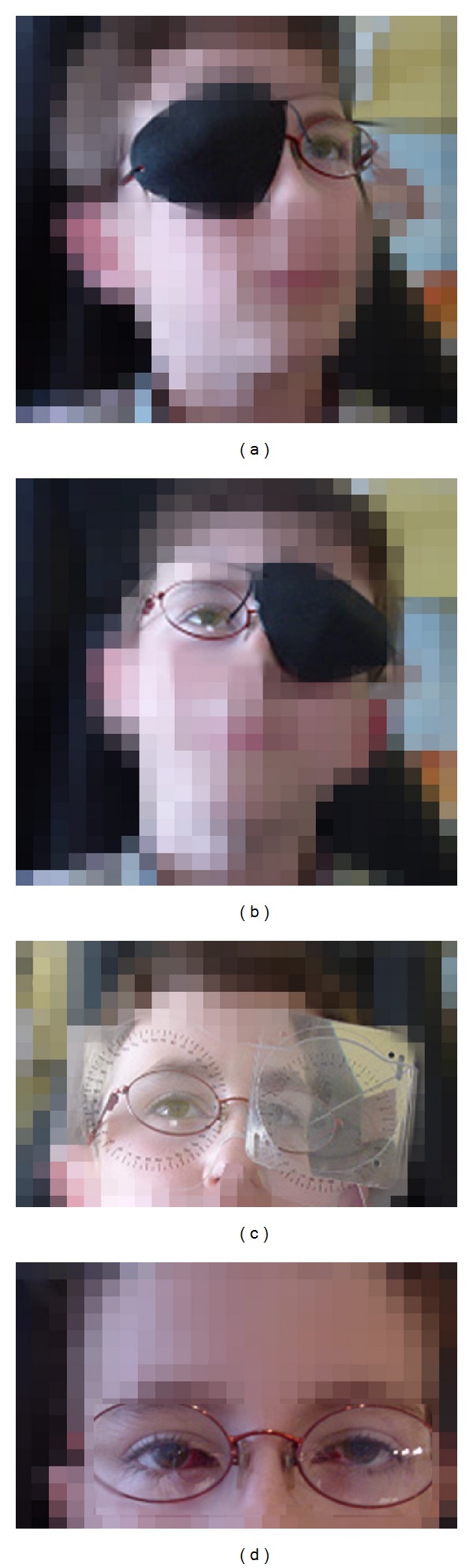
Discordant head turn and tilt induced by his left dominant eye (a and b). Base-out prisms are tested and the abnormal head position improved (40 diopters) (c). Therefore, a 5.5 mm medial rectus recession was performed and the torticollis disappeared (d).

*Visual acuity* should be carefully tested at near and at distance with the appropriate refraction. At distance, it must be measured for each eye as usual but also with both eyes open, since this situation represents a fixating effort, to determine the existence and the type of abnormal head postures adopted by the patient. The position with both eyes open is under the influence of the dominant eye. Testing at distance with one eye covered will help to distinguish between the concordant head turn, for example, one eye fixating in abduction and the other fixating in adduction maintaining the same head turn position with either eye covered; or the discordant nystagmus (Figure 1), in which abnormal head position changes depending on which eye fixes. The most common is the discordant head turn: each eye fixates in adduction [7]. Best visual acuity should be also tested in the null position, because if it is only assessed in primary position, visual acuity could be underestimated [8]. Spielmann also advocates testing not only visual acuity at near, but the distance at which blocking convergence occurs.

The effect of *convergence* on nystagmus has to be carefully evaluated. If nystagmus decreases at near fixation, prism adaptation determines the largest amount of prism-induced convergence that decreases the nystagmus without creating diplopia [7].

The *head position* should be noted according to the axis since it can be anomalous horizontally (right or left head turn), vertically (chin up or down), torsionally (right or left head tilt), or in a mixed pattern (Figure 1). Each of the components can be measured using an orthopedic goniometer or a sensitive torticollometer placed on the patient's head [10]. It is very useful to evaluate the family photo album [8]. The nystagmus amplitude may be so small as to be detectable only with the aid of a slit lamp. Therefore, if a patient presents an abnormal head posture, a thorough examination should be conducted in order to determine whether a very small amplitude nystagmus may underlie the head tilt [8].

The *prisms* are very useful to assess the possible outcomes of surgical treatment for nystagmus (Figure 1), even though they can be used therapeutically too in the mildest cases. Abnormal head posture can be quantified according to the amount of prismatic diopters needed to move the image to a centered null zone. In mixed nystagmus, the attenuation of additional components when base prisms are placed horizontally may indicate us that only the right or left head turn must be surgically corrected. Base-out prisms can be used to determine if the nystagmus decreases with convergence and to simulate divergence surgery to predict its usefulness to improve visual acuity or head posture (Figure 1). Besides, results can be compared by simulating different surgical approaches with prisms in patients with abnormal head posture and blockage in convergence, in order to decide between an artificial divergence surgery or a yoke muscles recession, for example. Up and down chin torticollis are frequently due to A and V syndromes, respectively, so the abnormal head posture looks for the zone at which convergence is needed. Therefore, horizontal divergence surgery may be the best option for these cases. This comparison may be more accurately performed assisted by electronystagmography or videonystagmography, which can measure the effects of prisms on nystagmus intensity.

## 5. Treatment

The objectives in the treatment of patients with nystagmus are as follows [11]:

- to try to improve visual acuity by diminishing the amplitude and frequency of nystagmus movements,
- to transfer the nystagmus blockage position from an extreme position to a frontal one, in order to improve abnormal head position,
- to correct strabismus when it is present.

### 5.1. Correction of Refractive Errors

The first step of management is the correction of any present refractive error. The prevalence of ametropia is much higher in patients with nystagmus than in the general population [12]. Almost half of the children with congenital nystagmus also have astigmatism, which may not be present at birth, but may appear during the first decade of life [13]. Our group analyzed the refractive error in 81 children with nystagmus (data not published) and found myopic astigmatism to be the most prevalent type (43% of eyes). The head turn may make children to avoid looking through the optic center of the glasses, and therefore, some authors consider contact lenses a better option to correct astigmatism in these children.

### 5.2. Prisms

If the null zone is fairly close to the primary position, yoked prisms may be used in spectacles to shift the image centrally. Based-out prisms can be used to simulate artificial divergence surgery in patients whose nystagmus decreases with convergence. In prepresbyopic patients, it is important to combine them with −1 D lenses added to the patient's correction to overcome the effects of accommodation caused by convergence [8].

### 5.3. Medical Approach

A randomized study compared the use of gabapentin or memantine versus placebo in patients with secondary and idiopathic congenital nystagmus. Both GABA analogs showed a positive effect in visual acuity, reduced nystagmus intensity, and improved foveation, although the effects were weaker in those cases with afferent visual defects [14]. If this therapeutical option is chosen, treatment should commence with up to 2400 mg gabapentine per day in three divided doses. If no improvement is noted, then 20–40 mg of memantine may be prescribed [6]. Acquired periodic alternating nystagmus may improve when it is treated with baclofen [15]. However, the congenital form appears to be less responsive [6].

### 5.4. Botulinum Toxin

Botulinum toxin is a potent neurotoxin that blocks the release of acetylcholine at the neuromuscular junction of cholinergic nerves. Oleszezynska-Prost reported a decrease of amplitude of nystagmus and an improvement of visual acuity in 43% and 50% of patients with congenital nystagmus and esotropia or exotropia, treated with intramuscular injections of botulinum toxin. Anomalous head posture also improved. This approach was less useful in children with horizontal and vertical nystagmus [16]. Carruthers injected botulinum toxin in the horizontal rectus muscles of four patients with congenital nystagmus with improvement in visual acuity in three of them. The main limitation of this procedure is that the injections have to be repeated every 3 to 4 months [17].

Retrobulbar injection of botulinum toxin also has been reported to decrease the nystagmus and even to improve visual acuity in up to 66% of patients [18, 19]. Secondary strabismus, ptosis, or diplopia are possible transient side effects.

### 5.5. Surgery

**Figure 2:**
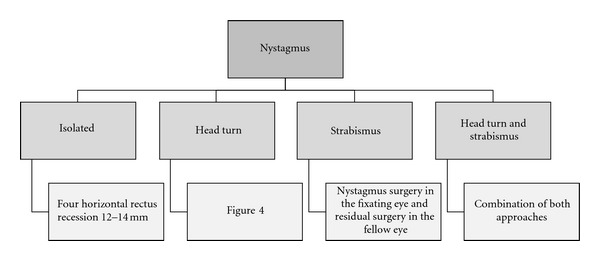
Our surgical approach to nystagmus depending on the associated features.

Figure 2 shows our surgical strategy for nystagmus.

#### 5.5.1. Surgery to Decrease Nystagmus

Hertle analyzed available data on surgery for nystagmus and concluded that 85% of the surgical procedures aim to improve the nystagmus and the anomalous head or eye position. However, visual function improves with eye muscle surgery because patients recognize objects faster and have less head movement, better motion, and contrast sensitivity. The current hypothesis is that in surgical interference with peripheral extraocular tendons, proprioceptive nerve endings influence central ocular motor pathways, resulting in an improved oscillation [20].

**Figure 3:**
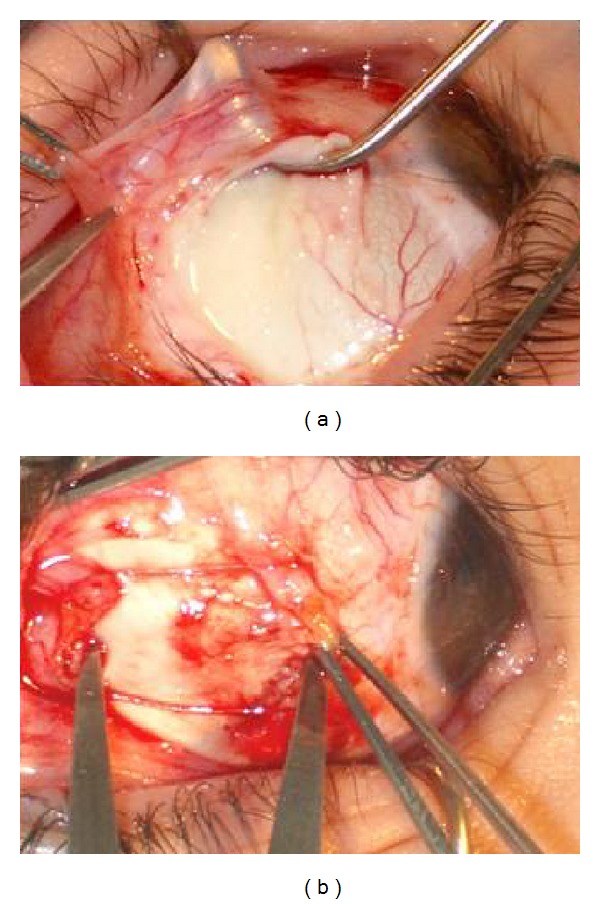
Large lateral rectus reccesion.

Initial surgical procedures aimed to lessen muscle efficiency and to reduce nystagmus intensity in order to improve visual acuity. Tenotomies, miotomies, or faden operation of the four horizontal rectus muscles have been tried [7]. The most useful method according to our experience [21, 22] is the retroequatorial recession of the two horizontal muscles of each eye (Figure 3), as initially proposed by Rama, Bietti and Bagolini [23, 24]. Spielmann performed this surgery when no abnormal head posture is found since he considers that the absence of a head turn indicated an absence of compensatory mechanisms [7].

Large recessions of the four horizontal rectus muscles is useful in cases without abnormal head posture or in those with active blockage of nystagmus by convergence or in extreme lateral gaze. Bagolini et al. differentiated two types of head-turns due to nystagmus. The most common type is composed of patients whose head turn is explained with the null position of Kestenbaum in which the nystagmus disappears or is reduced. The second one is represented by patients who actively block the nystagmus, by means of an increased discharge of the extraocular muscles who are synergistic and responsible for the head turn. These patients use large head turns to place their eyes in extreme sidegaze, much more pronounced than head turns associated with a null zone, fixating in extreme adduction. The same mechanism of active blockage occurs when infantile nystagmus is decreased in convergence [25].

#### 5.5.2. Surgery for Patients with Compensatory Head Turns

The improvement of an abnormal head position is important to enlarge the visual field, to eliminate the possibility of later problems arising from long-term abnormal contracture of the neck muscles, and to permit an adequate vision through the center of the spectacles (mainly in subjects with high astigmatism, and also for cosmetic and social purposes) [7, 8].

**Figure 4:**
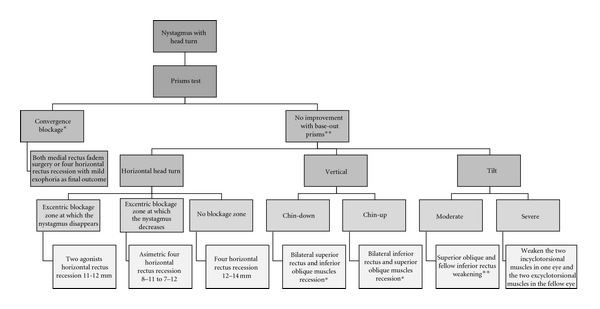
Our surgical approach to abnormal head posture associated to nystagmus. In patients whose nystagmus decreases with convergence, base-out prisms are tested and if the nystagmus shows significant improvement or disappears, an artificial divergence surgery is proposed. Remember that this strategy is frequently useful in discordant nystagmus and in vertical head turns. Otherwise, a surgical procedure of the horizontal muscles may be simulated with prisms because it could suffice in any type of abnormal head turn. Finally, pure vertical or torsional abnormal head positions may seldom need surgery on the vertical rectus and/or oblique muscles. In children under two years of age, botulinium toxin injected in the retrobulbar space* or in the muscles** is preferred to surgery.

Figure 4 shows our group's surgical strategy for a head turn associated to nystagmus.

**(1) Horizontal Head Turn**

Kestenbaum and Anderson were the first-to-perform procedures aiming to shift both eyes from the null zone into the primary position. Anderson proposed to weaken the horizontal rectus muscles that are activated during the slow phase of the nystagmus because they were thought to have a greater tone than their antagonists. Therefore, in a patient with a concordant left head turn (the left eye fixing in adduction and the right eye in abduction), an Anderson-like procedure would consist of a supralarge recession of the right lateral rectus and left medial rectus muscles [7, 26]. A Kestenbaum [27] procedure combined 5 mm recessions with resections of the antagonist muscles; however, the initial procedure led to a high rate of hypocorrections and Pratt-Johnson increased the amount of muscle surgery. The former 5 mm recessions and resections were increased to 10 mm [28].

Parks modified the Kestembaum technique to the 5-6-7-8 or the “Classic Maximum” procedure in which a recession of 7 mm was performed to the lateral rectus, a resection of 6 mm to the medial rectus of the abducted eye, a recession of 5 mm to the medial rectus, and finally a resection of 8 mm to the lateral rectus of the adducted eye [29]. These amounts were increased later and the procedure was named “Classic Plus” [30].

New or recurrent compensatory head postures can reappear years after the initial treatment, and further surgery may be necessary [31].

**(2) Vertical Head Turn**

Parks proposed performing a recession of the vertical rectus agonists in those cases in which the head turn was less than 25°, and to add a resection of the other two vertical rectus in more severe cases [29]. The same idea may be applied for vertical head postures. When nystagmus is absent in downward gaze, which induces chin elevation, the eyes should be surgically shifted upward by recessing both inferior rectus muscles. Head tilts are the consequence of a compensatory cycloversion [7]. However, some vertical head turns are due to exotropia/phoria at up or down gaze, at which the convergence mechanism is activated decreasing the nystagmus. The prisms tests are useful to discriminate between the most useful surgical approach, a vertical rectus and oblique muscles surgery, or an artificial divergence surgery.

**(3) Head Tilt**

In a right head tilt, we should balance the incyclotorsion of the right eye and the excyclotorsion of the left eye [7]. The former could be obtained either by a superior oblique tenectomy or a recession of two thirds of its anterior fibers; or by reinforcing the inferior oblique. Both techniques may be combined. To balance the excyclotorsion of the fellow eye, the two thirds of the anterior fibers of the superior oblique could be advanced and/or the inferior oblique could be weakened by myectomy of the two thirds of the anterior fibers and recession of the posterior fibers [32]. If vertical deviations are induced, vertical rectus surgeries may be performed later.

**(4) Mixed Abnormal Head Position**

Abnormal head position may be also mixed. Arroyo-Yllanes et al. treated 21 cases with only horizontal surgery using a modified Anderson procedure: a recession of the muscles responsible for the compensatory head position, which are fixated 2 mm posterior to the equator and additional surgery for strabismus correction whenever needed. All but one patient showed improvement in the head turn, even though five developed mild overcorrection. In all but one case, the results remained stable throughout the followup. Therefore, head tilt and vertical head turns may improve with only weakening surgery of the horizontal rectus muscles in those cases in which the horizontal head turn predominates, at least when other components are not severe. This approach is thought to work by moving the blockage point to the primary position where the cyclovertical muscles' actions are weaker [11].

#### 5.5.3. Nystagmus and Strabismus without Head Turn

Nystagmus surgery should be performed in the fixating eye and residual strabismus assessed in the fellow eye. For example, in a patient with esotropia and right eye fixation, a 12 mm recession would be done in both horizontal rectus of the right eye and in the left medial rectus associated to a variable amount of recession of the left lateral rectus depending on the strabismus severity.

#### 5.5.4. Nystagmus, Head Turn, and Strabismus

The previously reported surgical techniques should be combined as appropriate.

#### 5.5.5. Artificial Divergence Surgery

The aim of the treatment is to create a supplementary convergence effort at distance that should decrease the nystagmus in those cases with blocking convergence. Spielmann performs bilateral medial rectus muscle recession ranging from 5 to 13 mm depending on the predetermined amount of fusion found with the prism test [7]. Bagolini et al. recommended generous recession and resection operations with addition of a Cüppers posterior-fixation suture to the recession of the lateroversor muscles responsible for head turn [25].

According to Abel, medial rectus recession should only be performed in those cases with convergence null without a concomitant gaze null [8]. In other cases, postoperative exotropia may be the aim of the head turn surgical strategy. The only exception is the nistagmus with discordant head turn since for most cases there is an associated blocking convergence permitting artificial divergence surgery (Figure 1) [7].

Artificial divergence surgery is only suitable in those cases in which we have confirmed the presence of fusion and stereoacuity in the preferred head position, and after measuring the fusional convergence amplitudes by placing base-out prisms before the eyes.

Bimedial recessions, Cüppers' faden operation of both medial rectus or the hemi-Kestembaum procedure are the main artificial divergence methods. The latter consists of applying the recession-resection Kestembaum principles only to one eye, the one that is in adduction in binocular vision.

The abnormal head position associated to nystagmus has to be corrected in many patients to improve their quality of life. However, it has to be carefully characterized to select the best surgical option.
